# Landscape simplification filters species traits and drives biotic homogenization

**DOI:** 10.1038/ncomms9568

**Published:** 2015-10-20

**Authors:** Sagrario Gámez-Virués, David J. Perović, Martin M. Gossner, Carmen Börschig, Nico Blüthgen, Heike de Jong, Nadja K. Simons, Alexandra-Maria Klein, Jochen Krauss, Gwen Maier, Christoph Scherber, Juliane Steckel, Christoph Rothenwöhrer, Ingolf Steffan-Dewenter, Christiane N. Weiner, Wolfgang Weisser, Michael Werner, Teja Tscharntke, Catrin Westphal

**Affiliations:** 1 Agroecology, Department of Crop Sciences, Georg-August-University Göttingen, D-37077 Göttingen, Germany; 2 Terrestrial Ecology Research Group, Department of Ecology and Ecosystem Management, Center for Food and Life Sciences Weihenstephan, Technical University of Munich, D-85354 Freising, Germany; 3 Ecological Networks, Biology, Technical University of Darmstadt, D-64287 Darmstadt, Germany; 4 Nature Conservation and Landscape Ecology, Institute of Earth and Environmental Sciences, University of Freiburg, D-79106 Freiburg, Germany; 5 Department of Animal Ecology and Tropical Biology, University of Würzburg, Biocenter, D-97074 Würzburg, Germany

## Abstract

Biodiversity loss can affect the viability of ecosystems by decreasing the ability of communities to respond to environmental change and disturbances. Agricultural intensification is a major driver of biodiversity loss and has multiple components operating at different spatial scales: from in-field management intensity to landscape-scale simplification. Here we show that landscape-level effects dominate functional community composition and can even buffer the effects of in-field management intensification on functional homogenization, and that animal communities in real-world managed landscapes show a unified response (across orders and guilds) to both landscape-scale simplification and in-field intensification. Adults and larvae with specialized feeding habits, species with shorter activity periods and relatively small body sizes are selected against in simplified landscapes with intense in-field management. Our results demonstrate that the diversity of land cover types at the landscape scale is critical for maintaining communities, which are functionally diverse, even in landscapes where in-field management intensity is high.

## Abstract (web summary)

Agricultural intensification may negatively impact biodiversity via a number of mechanisms. Here, Gámez-Virués *et al.* show that landscape simplification acts as an environmental filter to homogenise grassland arthropod communities into pools of species with less specialised functional traits.

## Graphical abstract

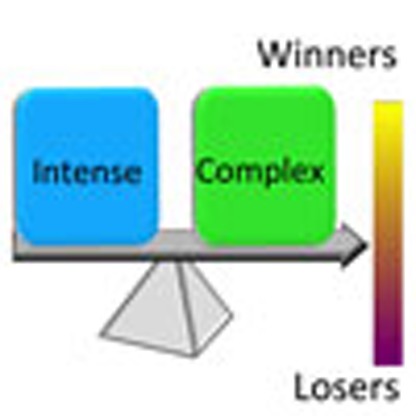

Agricultural intensification acts as an ecological filter^1^ that can eliminate entire communities in the process of biotic homogenization^2^,^3^,^4^,^5^—a shift towards communities with shared traits^6^—limiting the response-diversity and resilience of ecosystems against disturbances^2^,^7^,^8^. Greater numbers of functionally similar species in a community offer higher probabilities that at least some species will survive stochastic and deterministic processes, and maintain ecosystem functioning—as stated by the insurance hypothesis^7^—if these species show variation in their responses to environmental filters. Biotic homogenization that results in communities with reduced response diversity can limit the functions provided by the community, and their ability to respond to disturbances, leading to the deterioration of ecosystem goods and services^2^,^3^,^4^,^5^. Identifying shared functional traits (trait syndromes) common to species that respond in a similar way to the same environmental filters can elucidate how these filters affect functional community composition^9^. Identifying these trait syndromes can also identify species at risk, because such effects can be detected before species losses and extinctions occur^2^,^4^,^5^,^9^,^10^,^11^. Studies applying trait-based functional community approaches for animals are still developing. Traits such as dietary breadth, dispersal ability, life-history and body size have been studied individually to evaluate their role in the response of communities to agricultural intensification^2^,^4^,^5^,^10^,^11^,^12^,^13^. For example, species with limited dispersal ability, narrow dietary breadth and low fecundity have been identified as being at higher risk of extinction in intensively managed ecosystems^3^,^4^,^11^,^12^,^14^.

Although species loss and biotic homogenization^7^ have been identified as major responses to agricultural intensification—clearly evidenced by the decline of specialist species, progressively replaced by more-generalist species^3^,^4^,^10^,^11^,^12^—the effects of agricultural intensification at different spatial scales on functional community composition are unknown for almost all agroecosystems, but see refs 11, 13, 15. In central-European grasslands, for example, local (in-field) scale intensification—greater fertilizer input and greater frequencies of mowing and grazing^16^—has been shown to act as an ecological filter, leading to functional homogenization of indicator-species, such as butterflies^12^. Meanwhile, landscape scale simplification—both reduced diversity of land cover types (reduced compositional landscape heterogeneity) and an increase in patch sizes within the landscape (reduced configurational landscape heterogeneity)—has also been shown to act as an ecological filter, selecting against specialized butterfly species^11^(see refs 5, 13 for other variables). Landscape simplification has been hypothesized to strongly influence local patterns of species richness and abundance because of a reduced capacity to support a large species-pool and the lack of opportunity for spill-over between complementary resources^17^. Theoretically then, landscape-scale effects could dominate community-level filtering of functional traits (Fig. 1), because the negative effects of in-field management intensification are expected to be compounded by landscape-scale simplification, but buffered by landscape heterogeneity, which favours spill-over and supporting a larger species pool^17^.

The current understanding of the effects of landscape simplification on functional community composition of arthropods has not been extended to cover the diverse, functionally distinct taxa of comprising the arthropod community as a whole. Furthermore, the two distinct components of landscape simplification: compositional heterogeneity (diversity of land cover types) and configurational heterogeneity^18^ (size and arrangement of patches) have been overlooked by most landscape simplification studies, partly due to the difficulty in obtaining independent gradients of these two related, but independent, aspects of landscape simplification, but see refs 11, 18.

Here we identify complementary functional traits, at the community level, that are most sensitive to local-scale intensity (in-field management intensity^16^) and to landscape-scale simplification using independent gradients of composition (diversity of land cover types: measured using the Shannon diversity index) and configuration (average patch size within the surrounding landscape, that is, contiguous units of a single land cover type). Further, we disentangle these effects on the functional community composition for communities comprising multiple arthropod orders: an arthropod data set comprising multiple traits for 36,269 individuals from 598 species across Araneae, Coleoptera, Diptera, Hemiptera, Hymenoptera and Lepidoptera, ranging from soil dwelling decomposers, sessile and flying herbivores, cursorial and flying predators, to highly mobile mutualistic pollinators. All individuals were collected within a cohesive sampling effort from 72 managed grasslands across Germany. We use RLQ analysis^9^,^19^ to investigate the co-correlations between in-field management intensity and landscape-scale simplification (R table) and species trait attributes (Q table), constrained by the relative abundance of each species (L table) as observed in each of the 72 grasslands. RLQ analysis summarizes the co-correlation between the three tables (R, L & Q) into major correspondence axes (Fig. 2). The first axis defines the dominant co-correlation between different traits and environmental variables, and each successive axis summarizes the remaining co-correlation (Fig. 2). By examining the relative position of different species along multiple axes (biplots of the first two RLQ axes) and how they cluster together in this multi-dimensional space, we identify trait syndromes associated with different combinations of environmental filters^19^. Landscape variables were measured within two radii—500 and 2,000 m—because different species respond to landscape-scale processes at different spatial scales^1^,^17^ and these scales have different implications for management and policy decisions^18^. We hypothesize that in-field management intensity and landscape-scale simplification act as ecological filters, selecting against more-specialized fauna (Fig. 1a), and that landscape-scale effects dominate community-level filtering of functional traits (Fig. 1b). This is because the negative effects of in-field management intensification are expected to be compounded by landscape-scale simplification (decreased diversity of land cover types and increased average patch sizes), but be buffered by landscape heterogeneity (increased diversity of land cover types and decreased average patch size).

Our results demonstrate a unified response within the arthropod community to landscape-scale simplification and in-field intensification. Functional communities were homogenized and dominated by species with less-specialized feeding habits as larvae and adults, longer activity periods and relatively larger body sizes. Importantly, landscape-level filters, not local management intensity, dominate this process. Landscapes that remain less-simplified buffer the negative effects of in-field management intensity. Landscape structure is therefore much more important than previously realized in conserving biodiversity in managed landscapes and requires a management perspective and policy regulations to match.

## Results

### Landscape simplification dominates community composition

Landscape-scale filters dominated functional community composition at both spatial scales investigated. When considering landscape variables within the 500-m radius, the majority of co-inertia (that is, the strength of the association between traits and environmental filters, or how strongly these traits are filtered) was mainly represented by a strong association between configurational heterogeneity (average size of patches in the surrounding landscape), compositional heterogeneity (diversity of land cover types) and the traits representing adult and larval feeding breadth and activity period (Table 1; 1st axis). The trends indicate that specialized feeders (both larvae and adults) become less common as compositional heterogeneity decreases (lower diversity of land cover types) and configurational heterogeneity increases (smaller patches in the surrounding landscape), and also, partly, as in-field management intensity increases (Fig. 3a and Supplementary Fig. 1, from left to right, denoted by the direction of the red arrow). This association explains 91.7% of the co-inertia among the three RLQ tables (Table 1, 1st axis) at the 500-m spatial scale. The remaining variance (a further 7.1% of the co-inertia, 2nd axis) was explained by an association between in-field management intensity and configurational heterogeneity, and the relative body size (relative size within each order, see Methods for details; Table 1). These findings indicate that smaller species are less common when in-field management intensity increases and configurational heterogeneity decreases (larger average patch sizes; Table 1 and Fig. 3b from bottom to top).

The observed trends were consistent when landscape variables within a 2,000-m radius were analysed, but more numerous trait syndromes were detected (Fig. 4a and Supplementary Fig. 2). The dominant environmental filters determining functional community composition were again the configurational (average patch size) and compositional (diversity of land cover types) landscape heterogeneity, which strongly influenced adult and larval feeding breadth (feeding specialization). This association explained 88.0% of co-inertia among the three RLQ tables (Table 1, 1st axis at 2,000 m). However, secondary effects at this spatial scale (a further 10.5% of the co-inertia, 2nd axis) were associated with in-field management intensity and compositional landscape heterogeneity (Table 1, Fig. 4): an increase in both led to a strong reduction of relative body size (Fig. 4a, from bottom to top).

### Trait syndromes associated with agricultural intensification

RLQ biplots show the position of each species, and its particular combination of traits, along each of the three environmental filters concomitantly (Figs 3a and 4a). By examining the common traits, shared by species under different environmental conditions, we identified trait syndromes associated with the environmental filters, or combinations of filters. At the 500-m radius, species clustered into three major groups in terms of trait filtering (Fig. 3). Two of these clusters appear to be most strongly determined by feeding specialization for both adults and larvae (Fig. 3a, clusters A and C) and occur on the right-hand side of the RLQ axis where compositional heterogeneity is low, configurational heterogeneity is high and management intensity is high (as indicated by the direction of red arrows). Both clusters are composed almost entirely of generalist feeders (Fig. 3b–c). The third cluster (Cluster B), on the other hand, which is composed almost entirely of specialized feeding adults and larvae (Fig. 3b–c), occurs only where the landscape compositional heterogeneity is high (high diversity of land cover types), configurational heterogeneity is low (larger average patch sizes), and management intensity is low (Fig. 3a, left-hand side). Species with shorter activity periods became less common as both the compositional and configurational landscape heterogeneity decreased, and in-field management intensity increased (Table 1), however, these effects did not influence the clustering of trait syndrome groups. In addition, relatively small species became less common as the compositional heterogeneity and local management intensity increased, and configurational heterogeneity decreased (Fig. 3a, from bottom to top; Table 1, 2nd axis at 500 m). This filtering was evident for generalist feeders (Fig. 3a, Clusters A and C), but not for specialist feeders (Cluster B). The response of relative body size was more evident when landscape heterogeneity was measured at the 2,000-m spatial scale (compared with 500 m) in the way that species of different body size clustered in RLQ space, as species clustered by body size for both generalist and specialist feeders (revealing five clusters along the vertical axis rather than three; Fig. 4).

## Discussion

Our findings demonstrate that landscape variables act as a strong filter of functional-trait diversity for arthropod communities within managed grasslands, and dominate over local effects. Further, the diversity of land cover types (compositional landscape heterogeneity) has more consistent filtering effects across spatial scales, than the average patch size (configurational landscape heterogeneity), underpinning the need for targeted landscape management.

The first objective of this study was to identify the relative importance of landscape heterogeneity (compositional and configurational) and in-field management on the functional community composition of arthropods within managed grasslands. As predicted, landscape filters were strong and buffered against intense in-field management practices. This was especially true for compositional landscape heterogeneity; lending support to the landscape-moderated insurance hypothesis^17^—which posits that complexity (higher levels of landscape heterogeneity) provides stability and insurance against changing environments. The second objective was to compare the relative importance of compositional and configurational landscape heterogeneity for filtering functional community composition in managed grasslands—as these two aspects of landscape heterogeneity have differing ecological- and management-related consequences^18^. In this regard, compositional landscape heterogeneity proved to be a stronger and more consistent filter of diversity, and may therefore be the better environmental variable to consider in management and policy directives.

**Figure 1:**
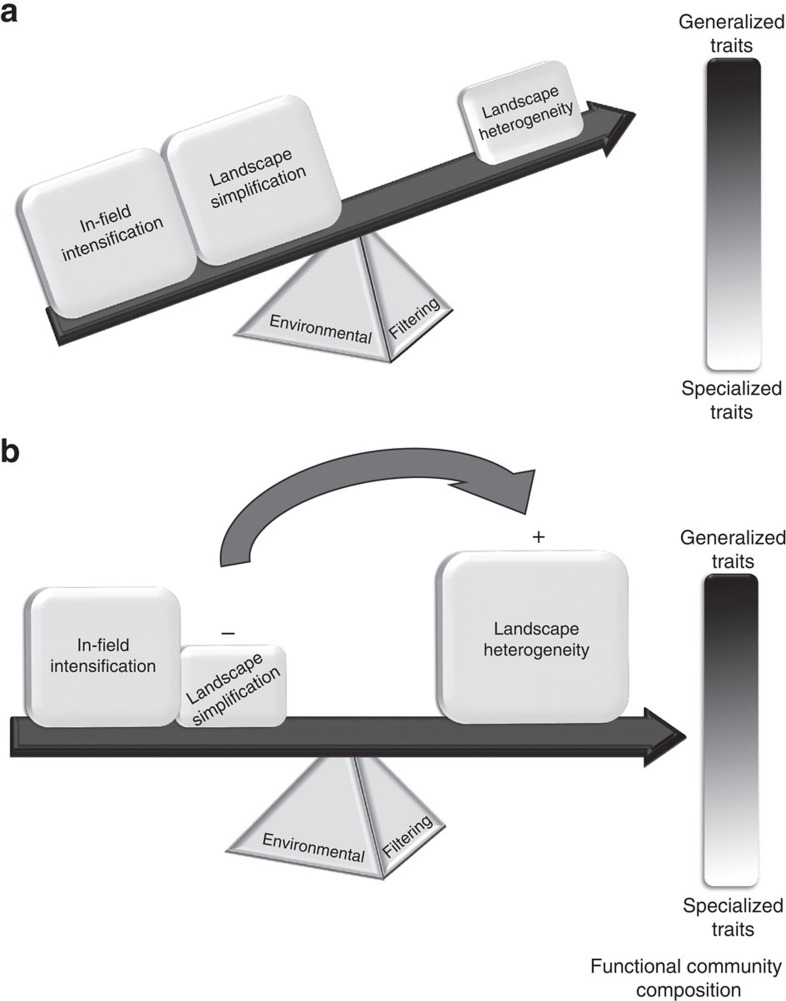
Functional traits as ecological indicators of intensification. High levels of in-field management intensity and simplification at the landscape scale select for a functional community comprising mainly species with generalized traits (**a**). Reducing simplification at the landscape-scale (−) by creating more landscape heterogeneity (**+**), in terms of high diversity of land cover types and small patch size, selects for a functional community comprising species with generalized and specialized traits, despite high levels of in-field management intensity (**b**).

The effects of compositional landscape heterogeneity on taxonomic diversity are well established^1^, however, evidence for the effects on functional community composition are still emerging^5^,^13^. Decreasing compositional heterogeneity at the landscape level can select against more-specialized species, leading to functional biotic homogenization, as shown in our study. Compositional landscape heterogeneity (measured as temporal stability: temporal turnover of land use type over a decade) was shown to strongly drive the loss of specialized birds across France, but configurational heterogeneity (measured as edge length) was equally significant^5^. Among carabid beetles, trait-syndromes were strongly filtered by compositional landscape heterogeneity (measured as proportional area of non-crop land)^13^, for example, small and very small carabid herbivores showed a strong association with decreases in compositional heterogeneity (high proportional area of non-crop land)^13^, whereas specialized predators, and to a lesser extent larger species of carabids, tended to be associated with landscapes with increased compositional heterogeneity dominated by grasslands^13^. However, other carabid species (most notably medium sized, generalized predators with dimorphic wings) were more strongly filtered by configurational heterogeneity (measured as cropland–woodland edges)^13^. In our study, compositional landscape heterogeneity was a stronger filter of functional community composition than configurational landscape heterogeneity, filtering feeding specialization along a diversity gradient of land cover types. Overall, species with generalist feeding adults and larvae, and with relatively large body sizes were favoured in simplified landscapes (those with fewer land cover types). Our measurement of compositional landscape heterogeneity considered the heterogeneity—the diversity of land cover types—as opposed to landscape-level disturbance (spatial^13^ or temporal^5^) tendencies, while we considered disturbance at the local scale. The ecological effects of compositional heterogeneity can vary between functional groups depending on whether composition is perceived as heterogeneity or disturbance^20^. At the community level, it appears that the complementary resources offered by habitat diversity strongly buffer the negative effects of the local disturbance regimes; in our study, specialized-feeding adults and larvae, were present only when the diversity of land cover types was high, even when in-field management intensity was high, as predicted (Fig. 1). These findings appear to support the landscape-moderated insurance hypothesis^17^, as landscape compositional heterogeneity provides insurance (resilience and stability) of functional traits in the face of intense management.

**Figure 3:**
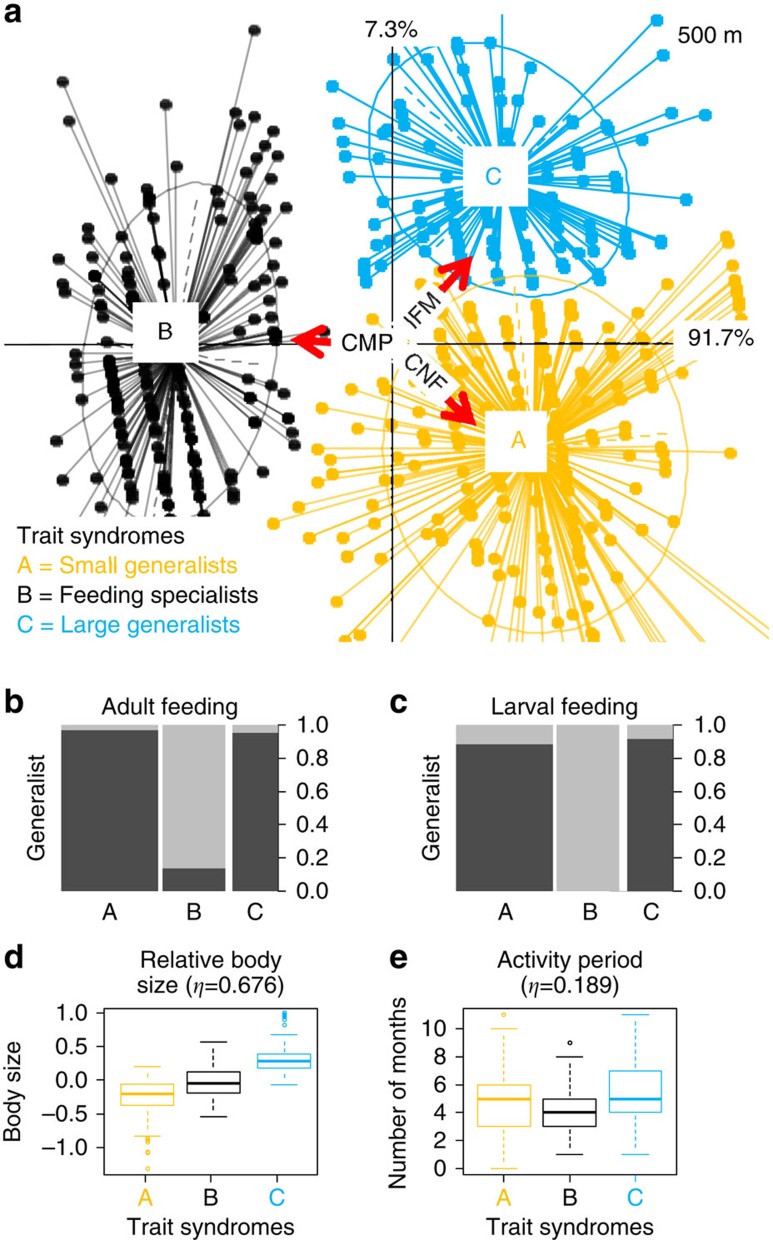
Trait responses to in-field management practices (IFM) and landscape variables (CNF=configurational landscape heterogeneity or patch size and CMP=compositional landscape heterogeneity or diversity of land cover types) within 500 m radius. RLQ biplot, showing the decomposition of co-correlations between environmental variables (R-Table 3 × 72) and trait attributes (Q-Table 598 × 4), constrained by abundance (L-Table 72 × 598). The size and direction of environmental effects are represented by red arrows. Clustered points identify trait syndrome groups and are represented with the same colour (**a**). Boxplots represent the distribution of trait attributes (mean, inner-quartile range (IQR) and outliers>1.5 × IQR) in cluster (**b**–**e**). The width of the bars represents relative abundance and the black component indicates the proportion of generalist feeders within the whole community: the higher the bar, the greater the proportion (**b**,**c**). Note: **a** has been rescaled for display purposes.

**Figure 4:**
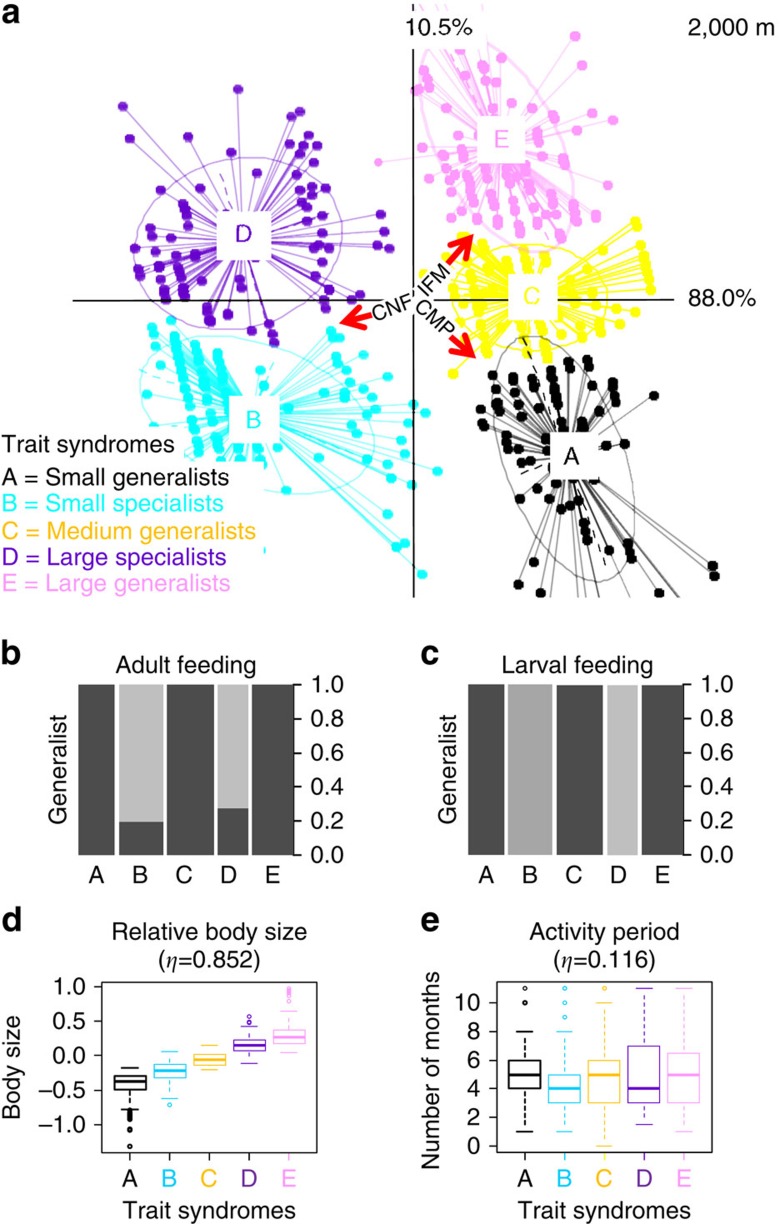
Trait responses to in-field management practices (IFM) and landscape variables (CNF=configurational landscape heterogeneity or patch size and CMP=compositional landscape heterogeneity or diversity of land cover types) within 2,000 m radius. RLQ biplot, showing the decomposition of co-correlations between environmental variables (R-Table 3 × 72) and trait attributes (Q-Table 598 × 4), constrained by abundance (L-Table 72 × 598). The size and direction of environmental effects are represented by red arrows (**a**). Clustered points identify trait syndrome groups and are represented with the same colour. Boxplots represent the distribution of trait attributes (mean, inner-quartile range (IQR) and outliers>1.5 × IQR) in cluster (**b**–**e**). The width of the bars represents relative abundance and the black component represents the proportion of generalist feeders within the whole community: the higher the bar, the greater the proportion (**b**,**c**). Note: **a** has been rescaled for display purposes.

In contrast to compositional heterogeneity, the effects of configurational heterogeneity on community composition were scale dependent. For example, at the 500-m scale, larger species were associated with larger average patch sizes (decreased configurational landscape heterogeneity), whereas at the 2,000-m scale, it was specialists feeders that were most strongly associated with larger patch sizes, even though such decreased configurational landscape heterogeneity is a symptom of landscape-level simplification, which may be expected to disadvantage specialist feeders. The different ecological processes that determine community composition occur at multiple spatial scales, for example, competition, carrying capacity and spill-over occur at a local scale, whereas dispersal occurs across larger scales^21^. Studies considering multiple spatial scales, such as ours, can offer an insight into the importance of these different ecological processes^22^,^23^, in particular, when interacting species respond to different spatial scales^1^. Configurational landscape heterogeneity has been suggested to be ecologically important as it may provide more opportunities for spill-over^18^,^24^—larger patches are surrounded by fewer neighbouring land cover types and offer less opportunities for spill-over. On the other hand, larger patches may be expected to have higher carrying capacity and to be able to support more species, or more larger species^25^. When we considered configurational landscape heterogeneity at the 500-m scale (that is, a 500-m radius surrounding the sampling point within the grassland), larger patches were strongly associated with body size, specifically with larger individuals. Contrary, compositional landscape heterogeneity (the diversity of land cover types) had no effect on body size at the 500-m scale (note that compositional landscape heterogeneity is pointing along the 1st axis in Fig. 3a, indicating no correlation with the 2nd axis and the relative changes in body size, from bottom to top). Suggesting that spill-over from complementary resources was not a factor in the response of body size at this spatial scale.

The size of patches has also been predicted to be important for specialized feeders, which have larger home-range requirements^26^,^27^. The strong response of specialist feeders to larger patches (reduced configurational landscape heterogeneity) observed in our study at both spatial scales maybe indicative of this phenomena. In addition, smaller patches may be more disruptive to dispersal at larger spatial scales. At the larger spatial scale (2,000 m radius), which is more reflective of the general landscape pattern, smaller patches within the landscape (increased configurational heterogeneity) were correlated with relatively larger insects—albeit only weak, this was the opposite trend from the 500-m spatial scale where the diversity of land cover types (that is, compositional landscape heterogeneity) was a stronger driver of body size. Larger, more mobile species, are perhaps responding to the higher diversity of land cover types, which represents more complementary resources available in the wider landscape^18^. The complex nature of the trait body size may also explain this scale-specific response to landscape heterogeneity. Body size is commonly used to reflect dispersal ability^28^,^29^,^30^,^31^,^32^,^33^. At larger spatial scales, larger body size may be expected to be related to smaller patch sizes, because larger species can disperse between smaller, more isolated, patches. However, the relationship between body size and dispersal has been shown to interact with other life-history traits in different ways for different taxa^30^,^31^,^34^,^35^. These complex interactions for body size among taxa may also explain the varying response emerging for body size to in-field management intensity^12^,^36^.

Managed landscapes are an important focus for biodiversity conservation as they represent more than a third of all land cover^37^. From a policy perspective, compositional and configurational landscape heterogeneity have different implications, both in terms of ecological benefits and also for management and marketing of production systems^18^. Hence, our findings demonstrate that landscape simplification, through reduced diversity of land cover types—currently ignored in management strategies—can select against more specialized species and that a decline in functional community composition can lead to functional homogenization. Thus, policy to prevent landscape simplification is warranted. Landscape management strategies that promote the maintenance of the diversity of land-uses (higher compositional landscape heterogeneity) are recommended for the conservation of a more diverse species pool. These strategies could also benefit government programmes in their efforts to reduce the loss of biodiversity in agricultural landscapes, for example, in the European Union^38^. Directives for configurational landscape heterogeneity are less clear cut and depend largely on the scale of management initiatives.

Finally, our trait-based approach, using multiple traits and multiple taxa, reveals the effects of agricultural intensification (at the in-field and landscape levels) on species with shared characteristics, and identify those trait syndromes that are prone to expansion or decline. Species with specialized feeding during their adult and larval stages, with shorter activity period, and with relatively smaller body size are more likely to be absent in intensively managed agroecosystems (large fields, high-intensity in-field management and low diversity of land cover types). Agricultural intensification, a type of human-induced disturbance, has contributed to the tremendous increases in food production over the past decades, but it has also altered the species pool, the patterns of resource availability and the interactions between them^39^,^40^. Consequently, the patterns of species distributions and functional community composition changed and caused mainly (but not entirely) the negative effects on ecosystem services and functions^1^,^3^,^4^,^5^,^11^,^12^,^13^,^41^. Although, many of these changes depend on the studied taxa and type of intensification^42^, general trends show a selection against more functionally diverse communities in agroecosystems with higher levels of in-field management intensity^3^,^4^,^5^,^11^,^12^,^13^. Our findings demonstrate that a reduction of intensification at the landscape scale can offset the effects of intense management of grasslands at the local scale and favour a more diverse functional community^43^. A more diverse functional community, in terms of functional traits, is more resilient to environmental changes, and can face disturbance without losing ecosystem functioning^3^,^7^,^8^,^44^. Maintaining functionally diverse communities should be a priority in sustaining the future^45^,^46^. As shown here, landscape-level processes can (i) dominate community assemblage in managed systems, especially for species susceptible to management intensity and (ii) buffer the adverse effects of high in-field management intensity, potentially contributing to a more sustainable agricultural intensification.

## Methods

### Study grasslands

Sampling plots were established within grasslands in three distinctive regions across Germany: (i) the UNESCO Biosphere Reserve Schorfheide-Chorin, a young glacial landscape, with numerous wetlands, situated in the lowlands of north-eastern Germany, (ii) the Hainich-Dün region including the Hainich National Park situated in the hilly lands of central Germany and (iii) the UNESCO Biosphere Reserve Schwäbische Alb, situated in the low mountain ranges of south-western Germany (Supplementary Fig. 3a). Each of these regions contains grasslands representative of the in-field management intensity utilized across Germany: ranging from hardly managed to highly fertilized and intensively used meadows and pastures^47^. Fifty experimental grassland plots (50 × 50 m^2^ each; hereafter referred to as grassland EPs) were established in each region (*n*=150) and carefully selected to cover the variation of in-field management intensity and soil depth found in each region while keeping consistency of soil type. Waterlogged sites and sites with a slope greater than 20% were excluded. From these initial 150 grassland EPs we selected sites for this study to represent (i) a wide gradient of in-field management intensity^47^ and (ii) independent gradients of compositional (that is, diversity of land cover types) and configurational landscape heterogeneity (that is, average patch size as an indicator of landscape connectivity) within the surrounding landscape (Supplementary Fig. 3b). In addition, grassland EPs where less than three individuals were recorded for a given taxonomic group were excluded. Further, six grassland EPs, which presented extreme values for one of the environmental variables (that is, average patch size), were also excluded (see below, under Landscape-scale variables). In total, our analysis included 72 grassland EPs, of which 27 were located in the Schorfheide-Chorin, 20 in the Hainich-Dün and 25 in the Schwäbische Alb (Supplementary Tables 1 and 2).

### Arthropod sampling

Arthropod sampling was conducted between May and September 2008. Araneae, Coleoptera, Diptera, Hemiptera (Auchenorrhyncha and Heteroptera) Hymenoptera and Lepidoptera were all collected within the 72 selected grassland EPs across the three regions.

*Araneae* and *Hemiptera (Auchenorrhyncha and Heteroptera)* were collected using sweep-netting^48^ with a total of 60 double-sweeps along three outer margins of each experimental plot (3 times 50 × 2 m^2^). All grassland EPs were sampled twice—two cover species with different phenologies: the first period in June and the second in August. Each sampling period covered a maximum of 2 weeks across regions. Specimens were stored in 70% ethanol and identified to species level with the help of experts (see Acknowledgements).

*Coleoptera* were collected using a D-Vac sampling device (Stihl SH 56) running for 1 min over a sampling covering 0.25 m area of vegetation, located at least 15 m from the edge of the grassland EP^49^. Four samples were taken in each plot. Each region was sampled twice: the first one between May and July, and the second between August and September. All beetles were identified to species level with the help of experts (see Acknowledgements).

*Diptera (Syrphidae)* and *Hymenoptera (Apidae)* were surveyed within a square transect along the four margins of the grassland EP (4 times 50 × 3 m^2^) between May and August^50^. Each survey covered a time span of 6 h between morning and afternoon. The transect was walked three times during one survey (three rounds, 2 h each) and all Syrphidae and Apidae that were observed visiting flowers were recorded. Species that could not be identified in the field were collected and identified to species level with the help of experts (see Acknowledgements).

*Lepidoptera* were surveyed following a line-transect method in each of the grassland EPs between May and August^12^. The transect was in total 300 m long and consisted of six walking paths (each of them 50 m long) distributed as follows: four paths along the four margins of the grassland EP and two paths inside the plot as to form three rectangles of equal dimensions. Three surveys per grassland EP were conducted in a randomized sequence per region. Surveys consisted of a 30-min walk along each transect during which all individuals within 2.5 m of each side of the line and 5 m in front of the recorder were identified and counted. Individuals were identified to species level^51^ in the field unless the taxonomic identity was unclear, in which case, specimens were kept for later identification by dissection of genitalia in the laboratory.

### Arthropod traits

Trait information was compiled from the literature for all species identified in our study. Adult feeding breadth, larval feeding breadth, relative body size and activity period were used as response variables. Feeding breadth was categorized as *specialist* (feeding on species within one family) or *generalist* (feeding on species within more than one family). Activity period was recorded as the number of months for which adults are active. Body size was defined as *relative* body size, which was calculated as the log difference between actual body size and the community average within each order. By considering body size as a relative measure within each order the influence of taxonomic difference on the overall trends of the community response to intensification was reduced.

### In-field management practices

In-field management intensity was defined through the land-use-intensity index^16^, which incorporates fertilizer inputs, mowing intensity and grazing intensity. For each grassland EP *k*, the land-use-intensity index *L*_*k*_ is defined as the square root of the sum of the three variables, each standardized by the regional mean of that variable;


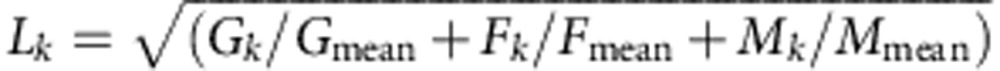


where *F*_*k*_ is the fertilization rate (kg N ha^−1^ per year), *M*_*k*_ is the mowing frequency (per year) and *G*_*k*_ is the stocking rate (livestock units d^−1^ ha^−1^ per year) for the plot, *G*_mean_, *F*_mean_ and *M*_mean_ are the respective regional-level means for fertilization rate, mowing frequency and stocking rate. We used the mean *L*_*k*_ for the years 2006–2008 as this best reflects the ongoing management intensity and has been shown to have a stronger effect on arthropod activity than single year indices^52^.

### Landscape-scale variables

Land cover features within 2 km of each grassland EP were recorded and mapped in the field in 2008. As patch borders did not vary between 2008 and 2009, polygon borders were defined using the latest high resolution (40 cm) aerial photographs from 2009 (ref. 53). Land cover features were classified in eight generalized categories (arable, forest, grasslands: managed grasslands and semi-natural vegetation, roads, trees: woodlots smaller than 1 ha, urban areas and water bodies; Supplementary Fig. 3b).

Landscape-scale simplification was represented by both compositional heterogeneity (measured as Shannon diversity of land cover types)^18^,^54^ and configurational heterogeneity (average patch size within the surrounding landscape)^18^,^54^. We chose Shannon diversity as a measure of compositional heterogeneity, rather than, for example, the proportional area of semi-natural habitats or non-arable land^55^, as our study includes many taxa that use and exploit a wide variety of different habitat types, and while Shannon diversity is highly correlated with the proportional area of arable fields in the landscapes, it displayed a wider range and a complementary independent gradient to landscape configurational heterogeneity. Both compositional and configurational heterogeneity were calculated at two spatial scales (500 and 2,000 m radii) surrounding the centre of each grassland EP. These two spatial scales were chosen to identify field-scale and landscape-scale effects. Shannon diversity of land cover types and average patch size were not correlated at the landscape scale of 500 m radius (*r*=0.360), and neither of them were correlated with in-field management (*r*=0.097; *r*=0.015, respectively). At the landscape scale of 2,000 m radius, the two landscape metrics were not correlated with each other (*r*=0.181) or with in-field management (*r*=0.118; *r*=0.031, respectively). Both landscape metrics were calculated utilizing user-defined work-flow tools in GIS-Software (ArcGIS 9.3, ESRI).

### Statistical analyses

Data from all sampling dates were pooled for each taxon and Hellinger transformations, using vegan package in R^56^, were applied to standardize abundance across taxa and to account for the long environmental gradients represented by the three regions^57^.

For RLQ analysis, the R- and Q-tables first underwent principle component analysis (the Q-table using the Hill and Smith method^58^ for mixing quantitative variables and factors) and the L-table underwent correspondence analysis. RLQ analysis was conducted using the ade4 package in R^19^.

Clusters within RLQ component-space were identified following Kleyer *et al.*^59^, based on Euclidean distances between species along the first two RLQ axes and clustered via Ward's hierarchical clustering, and Calinski–Harabasz stopping criterion^60^ to determine the optimal number of clusters. The degree of correlation between species-traits and response groups is expressed in correlation ratios. Analyses were conducted using R-codes adapted from those provided as Supplementary Material in Kleyer *et al.*^59^.

We tested for spatial autocorrelation in the co-inertia that was unexplained by each of the first two RLQ axes (the row scores—mR in the RLQ output—for each axis minus the total score for all axes) using the gearymoran test in the ade4 package (which uses neighbouring weights so that Moran's I and Geary's C randomization tests are equivalent)^61^,^62^. Spatial autocorrelation was not observed.

### Data accessibility

All data archived in BEXiS database, IDs: 17826, 16894, 16893, 4302, 3020, 15086.

## Additional information

**How to cite this article:** Gámez-Virués, S. *et al.* Landscape simplification filters species traits and drives biotic homogenization. *Nat. Commun.* 6:8568 doi: 10.1038/ncomms9568 (2015).

## Supplementary Material

Supplementary Information Supplementary Figures 1-3 and Supplementary Tables 1-2

## Figures and Tables

**Figure 2:**
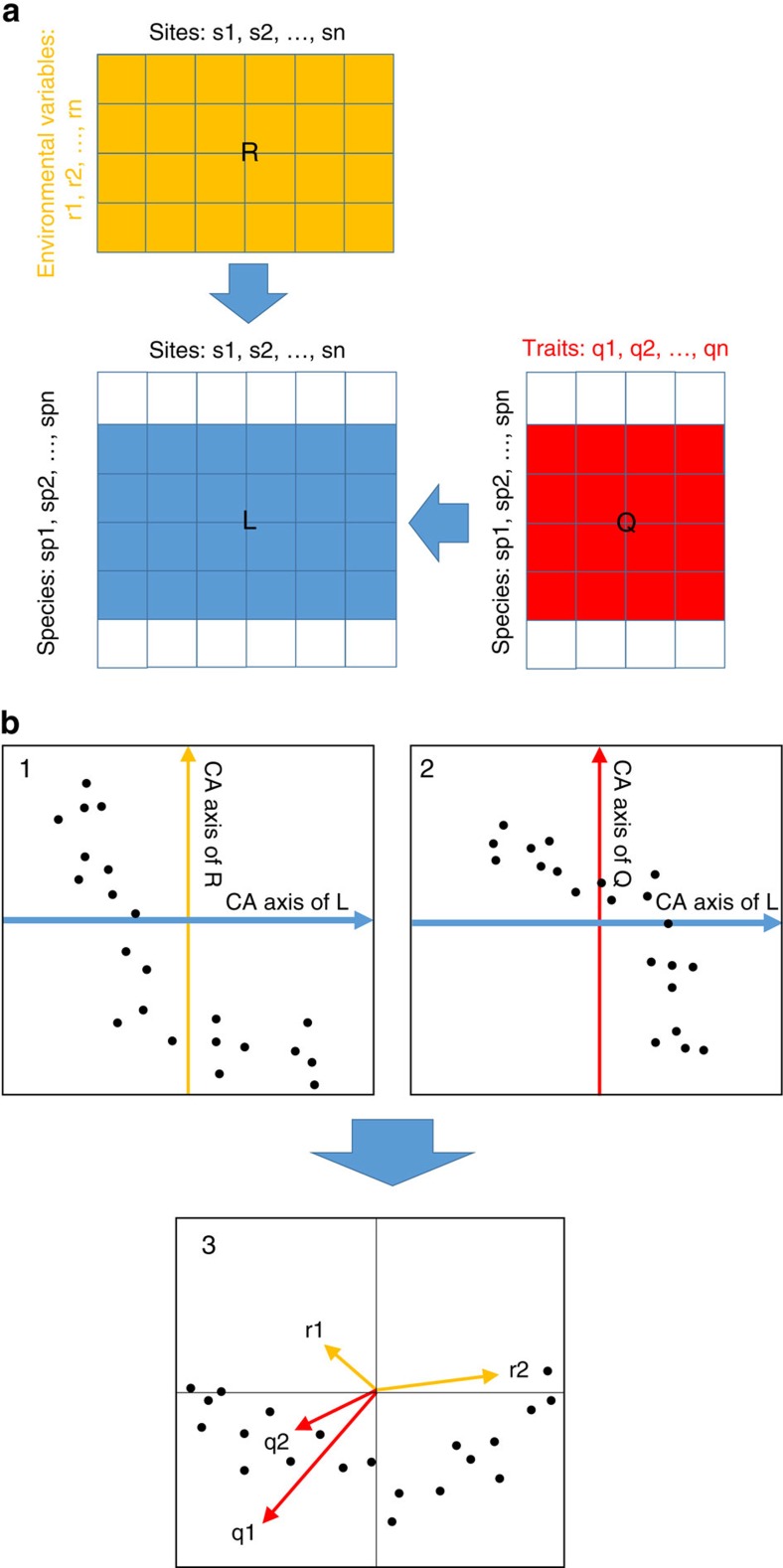
Conceptual overview of the RLQ analysis. RLQ is a co-inertia analysis that couples multiple data sets and identifies co-relationships between them. The multivariate RLQ analysis relates a species-traits table (Q) to a table of environmental variables at each site (R), using a species-abundances table (L) as a link (**a**). Correspondence analysis (CA) of the R variables against L (Plot 1) and Q variables against L (Plot 2) can be combined (Plot 3) to show the *r*-components (environmental variables from Table R) and *q*-components (species traits from Table Q) as vectors within a single bi-plot (**b**). Modified with kind permission from Dolédec *et al*., Figures 1 and 5 (ref. 19).

**Table 1:** Correlation coefficients for the relationship between environmental variables and traits with the first and second RLQ axes at the spatial scales of 500 and 2,000 m radii.

	**500 m**		**2,000 m**	
	**1st axis**	**2nd axis**	**1st axis**	**2nd axis**
*Environmental variables (d.f.=70)*				
In-field management intensity	0.346^*^	0.784^**^	0.334^*^	0.798^**^
Average patch size, ‘*configurational heterogeneity*'	−0.698^**^	0.630^**^	−0.604^**^	−0.128
Diversity of land cover types, ‘*compositional heterogeneity*'	−0.796^**^	0.358^*^	-0.769^**^	0.636^**^
				
*Traits (d.f.=596)*				
Activity period	0.422^**^	0.116^*^	0.303^**^	0.034
Adult feeding breadth	−0.832^**^	−0.007	−0.881^**^	−0.174^**^
Larval feeding breadth	−0.879^**^	−0.022	−0.905^**^	−0.184^**^
Relative body size	−0.170^**^	0.970^**^	-0.197^**^	0.973^**^

Bold *r*-values represent significant correlations.

*P<0.01.

^**^P<0.001.
