# Supplementary material for: Landscape simplification filters species traits and drives biotic homogenization
Source: Nat Commun. 2015 Oct 20;6:8568. doi: 10.1038/ncomms9568 (PMC4634213; doi:10.1038/ncomms9568)
Supplement: Supplementary Information — Supplementary Figures 1-3 and Supplementary Tables 1-2 [file ncomms9568-s1.pdf]

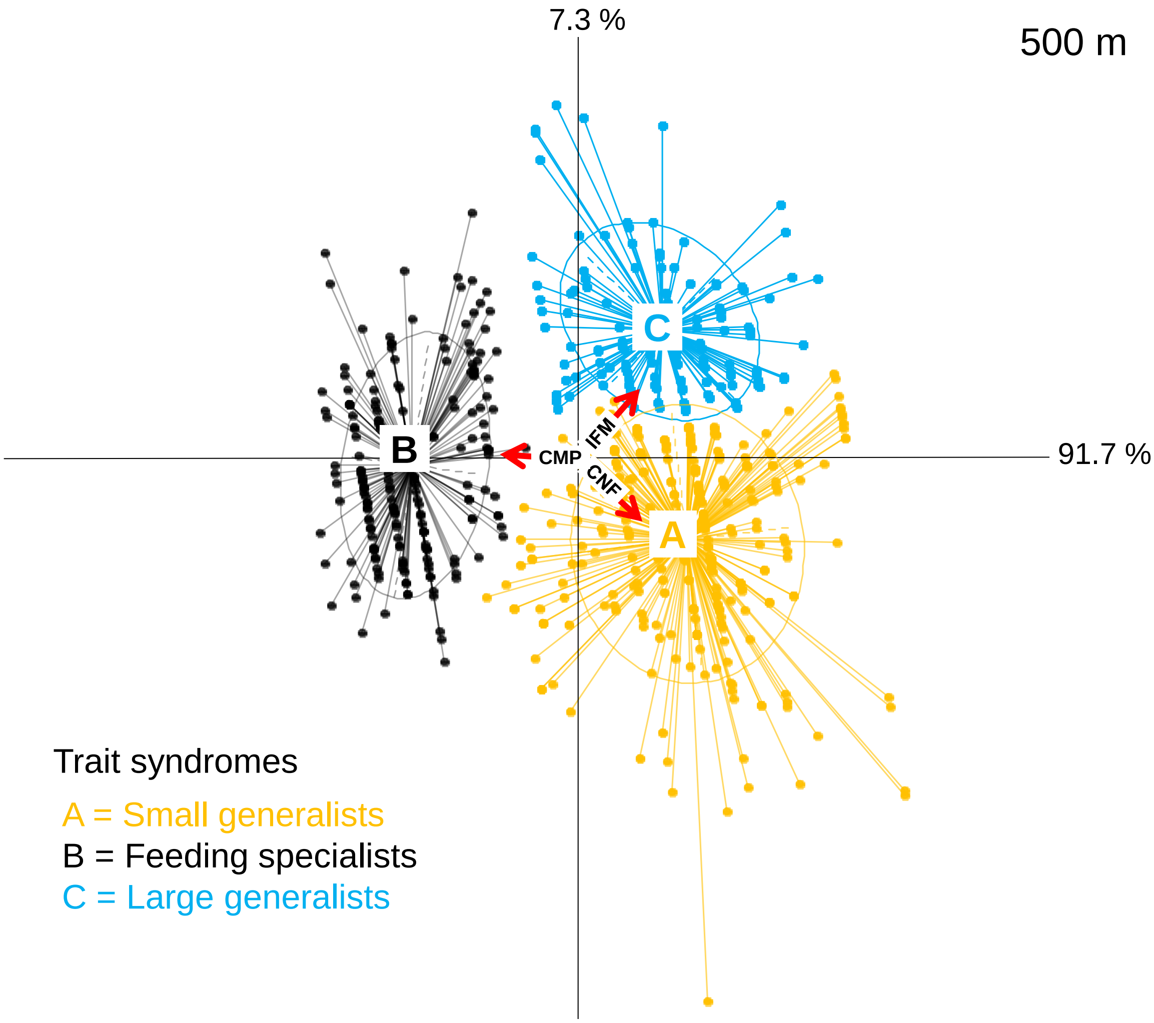

Supplementary Fig. 1 | Fullscale rendering of Figure 3 presented in main text. Trait responses to in-field management practices (IFM) and landscape variables (CNF=configurational landscape heterogeneity or patch size and CMP=compositional landscape heterogeneity or diversity of land cover types) within 500 m radius. RLQ biplot, showing the decomposition of co-correlations between environmental variables (R-Table 3x72) and trait attributes (Q-Table 598x4), constrained by abundance (L-Table 72x598). The size and direction of environmental effects is represented by red arrows. Clustered points identify trait sydrome groups and are represented with the same colour.

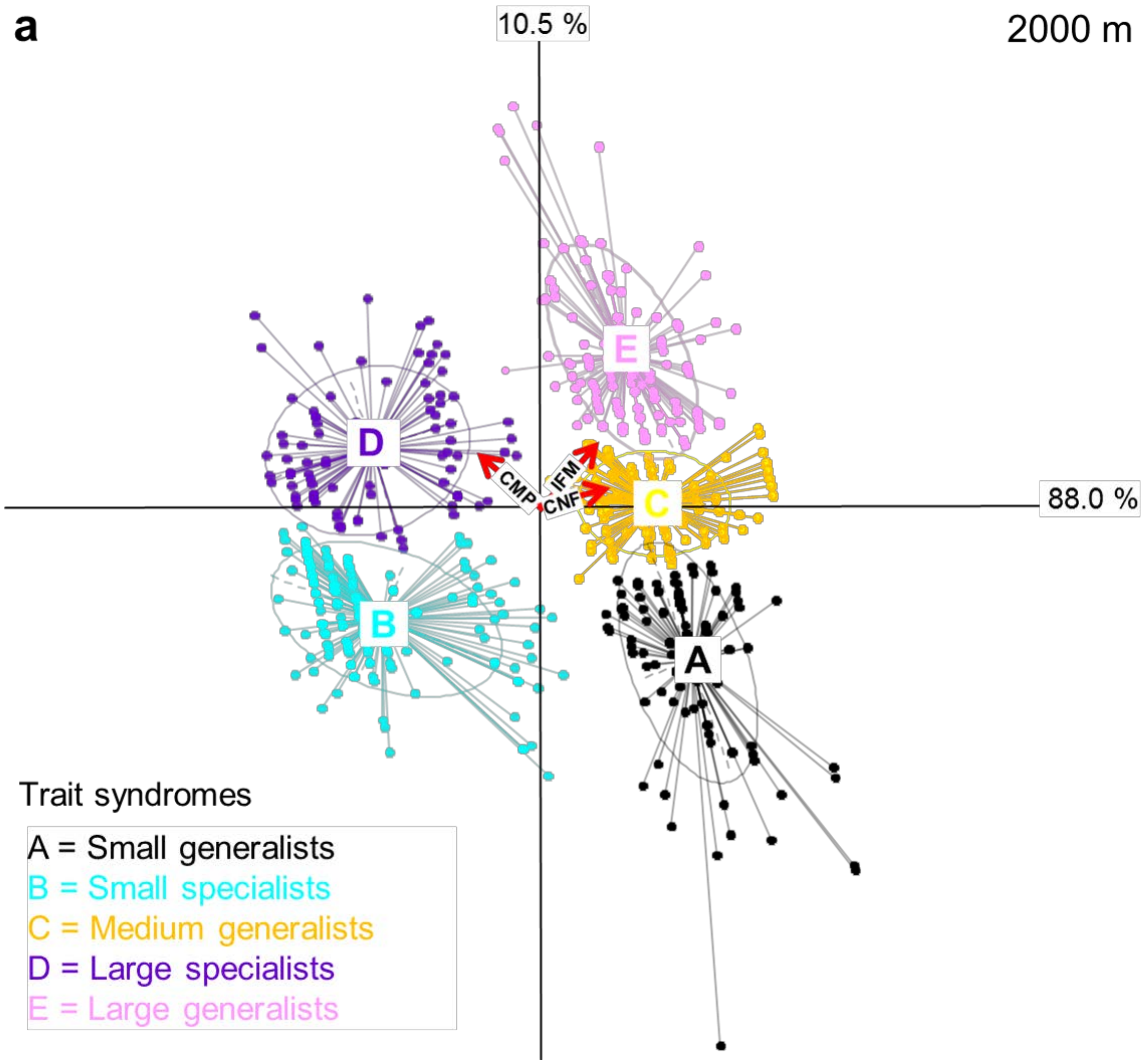

Supplementary Fig. 2 | Fullscale rendering of Figure 4 presented in main text. Trait responses to in-field management practices (IFM) and landscape variables (CNF=configurational landscape heterogeneity or patch size and CMP=compositional landscape heterogeneity or diversity of land cover types) within 2000 m radius. RLQ biplot, showing the decomposition of co-correlations between environmental variables (R-Table 3x72) and trait attributes (Q-Table 598x4), constrained by abundance (L-Table 72x598). The size and direction of environmental

effects is represented by red arrows. Clustered points identify trait sydrome groups and are represented with the same colour.

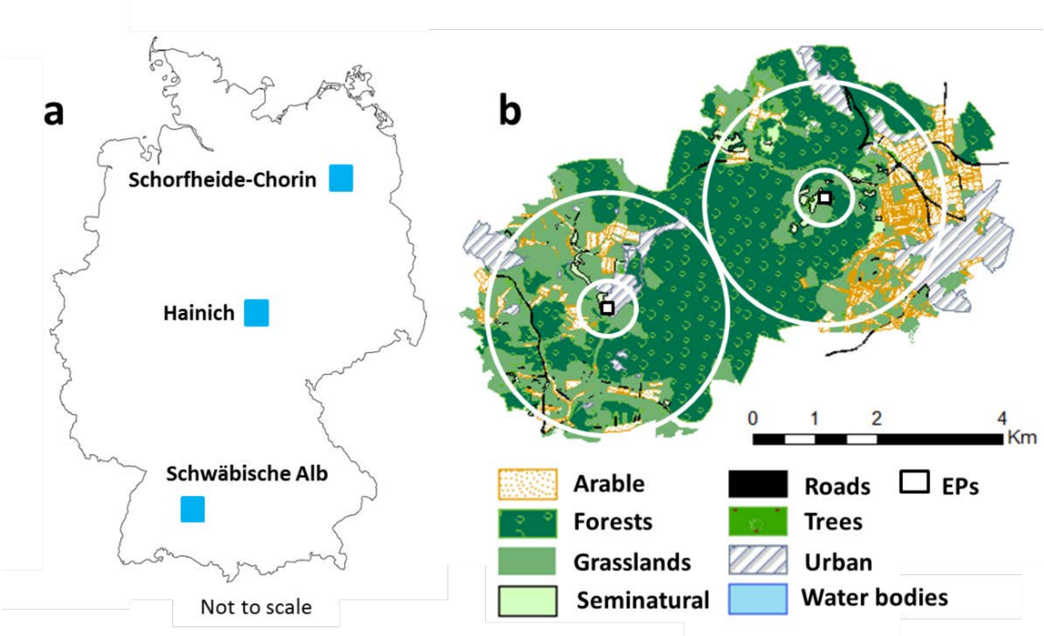

Supplementary Fig. 3 | Study regions and land cover type categories in landscapes surrounding grasslands. A total of 72 managed grasslands were sampled for arthropods across three regions in Germany (**a**). All land cover types surrounding each grassland EP (experimental plot) (**b**) were identified and mapped at 500 and 2000 m radii (represented with white circles) to calculate landscape metrics.

Supplementary Table 1 | Summary of distance between pairs of sites within each study region

Region	Distance (km)			% < 1 km*
	Minimum	Mean	Maximum	
Schorfheide-Chorin	0.270	12.520	28.110	6.7
Hainich-Dün	0.308	16.580	37.290	2.7
Schwäbische Alb	0.342	7.731	23.920	3.8

*Percentage of pairs of sites separated by less than 1 km

Supplementary Table 2 | XY-coordinates of the 72 selected grasslands (EP) (from 150 initial experimental plots) in the three regions. Values represent Gauss Krueger coordinates.

1. Schorfheide-Chorin			2. Hainich-Dün			3. Schwäbische Alb		
**EP**	**X**	**Y**	**EP**	**X**	**Y**	**EP**	**X**	**Y**
1	543110.0	588440.0	2	438990.0	565290.0	1	352540.0	536230.0
2	543180.0	588460.0	5	438290.1	567699.9	2	353510.0	536000.0
3	543220.0	588610.0	6	438769.8	567678.5	3	353949.0	536359.0
4	543330.0	588730.0	7	438918.0	568328.0	5	353260.0	536210.0
6	540789.9	588657.9	8	438970.0	568301.0	6	353278.0	536270.0
8	543432.0	588731.0	9	438699.1	567779.8	7	352798.5	536158.0
9	540720.0	588600.1	11	439239.0	568400.0	8	353650.0	536510.0
10	543300.0	588651.0	17	439290.0	566060.0	9	353731.0	536199.5
11	543269.1	588640.0	19	439310.0	566090.0	10	351990.0	536180.0
14	543181.0	588430.0	29	439530.0	568209.7	12	352601.5	536131.5
16	543312.1	588750.1	33	438987.9	566521.9	13	352690.0	536160.0
23	543490.0	588660.0	34	438739.7	567672.0	18	353870.0	536070.0
24	543288.0	588500.0	36	439560.0	565619.8	22	353809.0	536308.0
25	540759.0	588737.9	37	439590.1	565629.7	25	351930.0	536199.5
28	543355.0	588500.0	38	438397.9	566587.9	26	352998.5	536200.0
29	543305.1	588470.0	39	438431.9	566631.9	27	353560.0	536470.5
30	542190.0	589130.0	40	439100.0	564910.0	32	536990.0	536500.0
31	542222.0	589140.0	43	439102.0	568640.0	33	353640.0	536830.0
32	542200.1	589172.0	46	441299.8	567539.9	34	353710.0	536880.0
33	542240.0	587322.0	49	438790.0	568390.0	37	353070.0	536212.0
35	542278.0	587260.0				39	353160.0	536191.0
36	542207.5	587335.0				42	352792.0	536231.0

37	542491.1	588978.1	46	353228.0	536222.0
40	542251.0	588767.9	47	353310.0	536470.0
41	542330.3	588838.9	48	353698.5	536501.5
48	540680.1	588590.0			
49	542332.5	587197.5			
